# Renal Denervation: A Multicenter, Real-World Experience

**DOI:** 10.14740/cr2268

**Published:** 2026-08-31

**Authors:** Sarita Rao, Kamarishi Roshan Rao, Ajith Pillai, Amit Patil, Anuj Kapadiya, Arun Gopi, Gautam Swaroop, Harshal Ingle, Keyur Parikh, Nalin Kumar Mahesh, Nimit Shah, Praveen Chandra, Raj Pratap Singh, Ramesh Natarajan, Ravinder Singh Rao, Refai Showkathali, Shashi Mohan Sharma, Sunil Sathe, Sunip Banerjee, Varada Rajasekhar, Vithala Surya Prakasa Rao, Viveka Kumar, Yerramareddy Vijaychandra Reddy

**Affiliations:** a Apollo Hospitals, Indore, Madhya Pradesh, India; b Department of Cardiology, Apollo Hospitals, Indore, Madhya Pradesh, India; c Kauvery Hospital, Kovilambakkam, Chennai, Tamil Nadu, India; d Surana Sethia Hospital, Mumbai, Maharashtra, India; e AIG Hospitals, Hyderabad, Telangana, India; f Metromed International Cardiac Centre (MICC), Calicut, Kerala, India; g Tender Palm Hospital, Lucknow, Uttar Pradesh, India; h Ruby Hall Clinic, Pune, Maharashtra, India; i Marengo CIMS Hospital, Ahmedabad, Gujarat, India; j St. Gregorios Medical Mission Hospital (SGMMH), Parumala, Kerala, India; k Breach Candy Hospital, Mumbai, Maharashtra, India; l Medanta Hospital, Gurgaon, Haryana, India; m Graphic Era Institute of Medical Sciences, Dehradun, Uttarakhand, India; n KIIMS Health, Thiruvananthapuram, Kerala, India; o RHL Heart Centre, Jaipur, Rajasthan, India; p Apollo Main Hospitals, Chennai, Tamil Nadu, India; q Sawai Man Singh (SMS) Hospital, Jaipur, Rajasthan, India; r Poona Hospital, Pune, Maharashtra, India; s Kolkata Heart Lung Center, Kolkata, West Bengal, India; t Yashoda Hospitals, Hitech City, Hyderabad, Telangana, India; u Department of Cardiology, CARE Hospital, Hyderabad, Telangana, India; v Max Super Speciality Hospital, Saket, Delhi, India

**Keywords:** Renal denervation, Resistant hypertension, Blood pressure, India

## Abstract

**Background:**

Renal denervation (RDN) has emerged as a promising device-based therapy for the management of resistant hypertension (RH). However, real-world evidence of effectiveness and safety in the Indian population remains limited. This study aimed to evaluate the efficacy and safety of catheter-based RDN in reducing blood pressure (BP) and antihypertensive medication burden among Indian patients with RH.

**Methods:**

This retrospective, multicenter, real-world study included 26 consecutive patients who underwent RDN across 21 tertiary care centers in India. Patients were followed at discharge and at 1, 3, 6, and 12 months after the procedure. The primary endpoint was the change in office BP at 12 months. Secondary endpoints included changes in antihypertensive medication burden and procedure-related safety outcomes.

**Results:**

The mean age of the study population was 60.7 ± 13.8 years, and 61.5% were male. Baseline systolic blood pressure (SBP) and diastolic blood pressure (DBP) were 186.2 ± 18.8 and 98.7 ± 25.3 mm Hg, respectively. Significant reductions in BP were observed at all follow-up time points. At 12 months, mean SBP and DBP decreased to 121.7 ± 10.5 and 75.9 ± 4.7 mm Hg, corresponding to reductions of 63.4 and 24.7 mm Hg, respectively (both P < 0.001). The mean number of antihypertensive medication classes was significantly reduced from 5.12 ± 1.70 at baseline to 2.00 ± 0.80 at 1 year (P < 0.001). No major periprocedural complications or adverse events were reported. One patient experienced an access-site vascular complication involving the superficial femoral artery, which was successfully managed with covered stent implantation without further sequelae.

**Conclusions:**

RDN was associated with significant and sustained reductions in BP and antihypertensive medication burden over 12 months. The procedure demonstrated a favorable safety profile, supporting its role as an as a safe and effective adjunctive treatment option for patients with RH.

## Introduction

Hypertension is a major modifiable risk factor for cardiovascular morbidity and mortality worldwide [1]. In India, the ICMR-INDIAB-17 study reported a hypertension prevalence of 35.5%, corresponding to nearly 320 million affected adults [2]. Despite this substantial disease burden, national NFHS-5 data demonstrated that only 8.5% of hypertensive patients achieved optimal blood pressure (BP) control [3], reflecting major gaps in hypertension care and a potentially increasing burden of uncontrolled and apparent resistant hypertension (RH). Available Indian data estimate the prevalence of RH to range between 11% and 16.13% among patients with hypertension [4, 5]. Patients with RH are at substantially higher risk of adverse cardiovascular and renal outcomes, including all-cause mortality, myocardial infarction, heart failure, stroke, and chronic kidney disease, with an approximately 50% greater overall risk compared with patients without RH [6].

Timely recognition and appropriate treatment of RH are critical for improving BP control and reducing cardiovascular risk. Renal denervation (RDN) is a catheter-based therapy that reduces renal sympathetic nerve activity through radiofrequency (RF) ablation of the renal arteries. It has emerged as a promising adjunctive treatment option for patients with RH despite optimized pharmacological therapy [7]. Contemporary European Society of Hypertension (ESH) 2023 [8] and European Society of Cardiology (ESC) 2024 [9] guidelines recognize RDN as a validated intervention in appropriately selected patients, recommending its use within a structured, multidisciplinary care pathway.

Multiple randomized sham-controlled trials have demonstrated significant and durable reductions in BP with catheter-based RDN. However, major RDN studies including the SYMPLICITY, SPYRAL, RADIANCE trials and Global SYMPLICITY Registry programs, have been conducted predominantly in Western populations, with limited data available from India. Indian patients exhibit distinct clinical characteristics, including earlier onset hypertension, higher prevalence of diabetes and metabolic syndrome, increased salt sensitivity, and variable medication adherence patterns, which may influence response to RDN [10]. Therefore, the generalizability of existing RDN evidence to the Indian population remains uncertain, and real-world data from routine Indian clinical practice remain limited. The present study evaluated the real-world effectiveness and safety of catheter-based RDN in Indian patients with RH, with a focus on changes in BP and anti-hypertensive medication burden over 1-year follow-up.

## Materials and Methods

### Study design and study population

This was a multicenter, retrospective observational study evaluating the real-world use of catheter-based RDN in India during 2024–2025. Data were collected from 21 tertiary care centers, and a total of 26 consecutive patients who underwent RDN were included in the analysis. Given the retrospective design and absence of systematic ambulatory blood pressure monitoring (ABPM) and objective medication adherence assessment, the study population likely represented apparent treatment-RH.

Eligible participants were adults aged 18 years or older with RH, characterized by persistent uncontrolled BP despite treatment with at least three antihypertensive drug classes, including a diuretic, prescribed at stable or maximally tolerated doses for a minimum of 3 months. At baseline screening, eligibility required an office systolic blood pressure (SBP) of ≥ 140 mm Hg, calculated as the average of three seated measurements. Patients were excluded if they had secondary hypertension or more than one hospitalization for hypertensive emergency during the preceding year. Additional anatomical exclusions included renal artery stenosis exceeding 50%, renal artery aneurysm, previous renal artery intervention, multiple renal arteries, renal artery diameter < 4 mm, or an available treatable arterial segment < 20 mm.

### Study treatment and device

All patients underwent catheter-based RDN using the Symplicity™ renal denervation system (Medtronic, Minneapolis, MN, USA). The system consists of an RF generator and a single-use catheter designed to deliver controlled RF energy to the renal arterial wall for disruption of renal sympathetic nerves.

**Figure 1:**
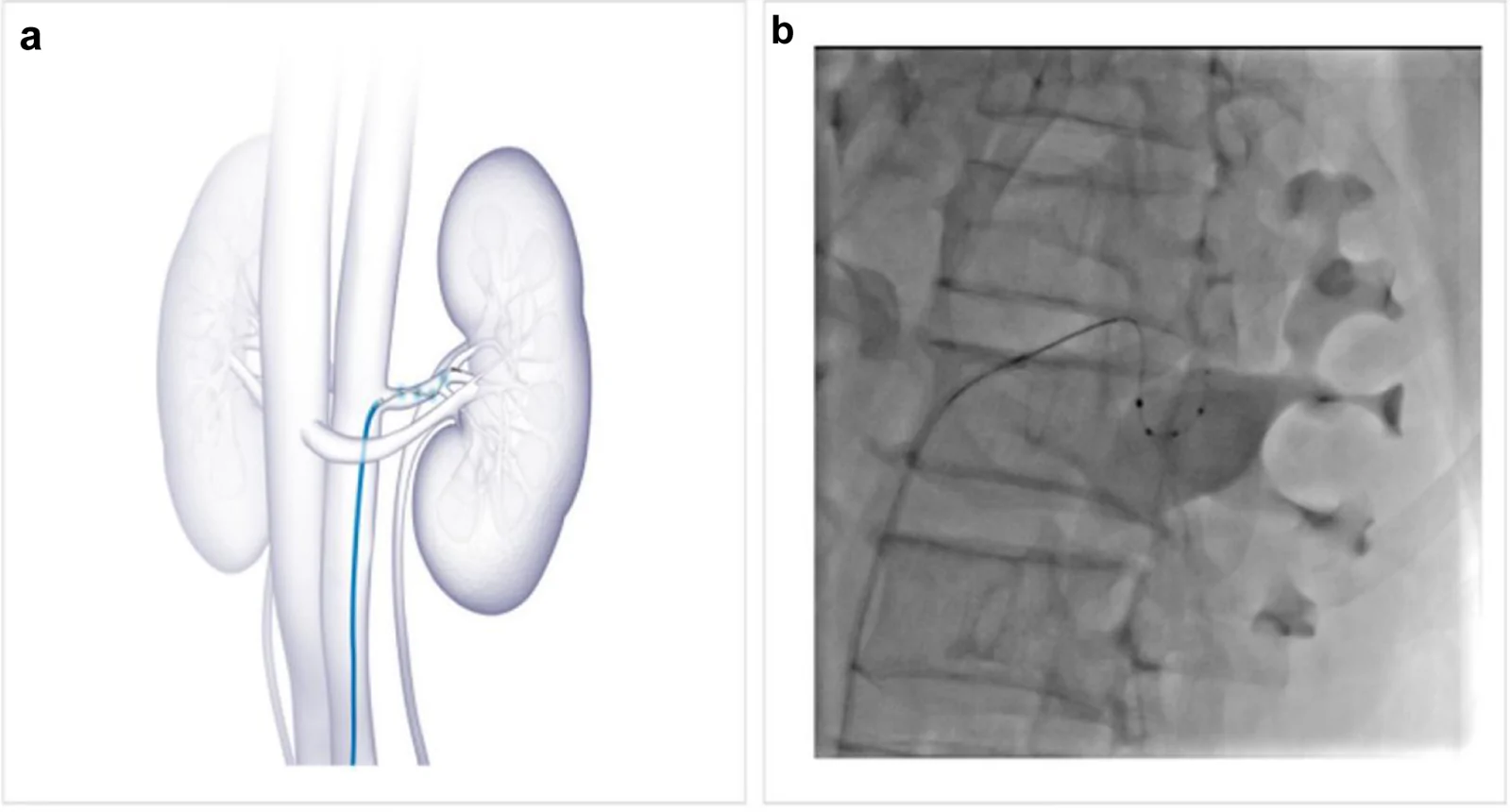
Catheter-based renal denervation. (a) Schematic representation of renal artery access for RDN. (b) Representative angiographic image during renal artery catheter positioning. RDN: renal denervation.

The Symplicity Spyral™ multi-electrode catheter was primarily used. It is a 5 Fr-compatible, over-the-wire device with a self-expanding nitinol spiral configuration incorporating four electrodes arranged circumferentially, enabling multi-point ablation within a single positioning. The Symplicity G3™ generator delivers automated RF energy using preset parameters with real-time monitoring of temperature and impedance (Fig. 1).

### Procedure

RDN was performed via percutaneous femoral arterial access under fluoroscopic guidance by experienced operators. Following renal angiography, the catheter was advanced into the main renal artery and its branches (3–8 mm diameter). A series of ablations were delivered in a helical pattern along the length of the renal arteries and accessible branch vessels, ensuring adequate longitudinal and circumferential coverage. Treatment strategy followed predefined protocols consistent with contemporary RDN techniques.

### Follow-up and assessments

Follow-up assessments were performed at 1, 3, 6, and 12 months after hospital discharge. BP measurements were obtained using standardized office-based techniques. At each visit, three consecutive BP readings were recorded after at least 5 min of rest, with 1-min intervals between measurements, and the average value was used for analysis. Anti-hypertensive medications were documented at baseline and during follow-up visits, with the number of medication classes recorded for analysis.

### Study outcomes

The primary outcome was the change in office BP from baseline to 1-year follow-up. Secondary outcomes included change in anti-hypertensive medication burden, assessed by the number of prescribed agents, as well as procedure-related complications during follow-up.

### Statistical analysis

Continuous variables were summarized as mean ± standard deviation, whereas categorical variables were reported as frequencies and percentages. Within-patient changes in SBP, diastolic blood pressure (DBP), and anti-hypertensive medication burden from baseline to each follow-up visit were assessed using paired Student’s *t*-tests. Subgroup analyses were performed descriptively, with paired Student’s *t*-tests used to evaluate changes from baseline within each subgroup. All tests were two-sided, and statistical significance was defined as P < 0.05.

### Ethical statements

The study was conducted in accordance with the ethical principles of the Declaration of Helsinki and was approved by the Kusum Independent Ethics Committee (No. PAPER_PUB/2026_01).

## Results

### Baseline characteristics

A total of 26 patients underwent RDN, with a predominance of males (61.5%) compared to females (38.5%). The mean age of the study population was 60.68 ± 13.83 years. The mean body mass index was 26.7 kg/m^2^. The mean duration of hypertension prior to RDN was 9.36 ± 7.71 years. Acute hypertension-related hospitalization was noted in 57.7% of patients, while 50.0% had at least one comorbidity. Renal Doppler evaluation was performed in 84.6% of patients, and anatomical complexity was noted in 19.2%. Procedurally, the mean duration of RDN was 79.61 ± 36.09 min. The mean total number of ablations performed was 27.48 ± 9.82 (Table 1).

**Table 1:** Baseline Clinical, Demographic, and Procedural Characteristics of the Study Population

Variable	Value
Gender	
Male	16 (61.5%)
Female	10 (38.5%)
Age (years)	60.68 ± 13.83
Weight (kg)	71.07
Height (cm)	163.15
Duration of hypertension prior to RDN (years)	9.36 ± 7.71
Smoking status	3 (11.5%)
Family history of hypertension	13 (50.0%)
Acute HTN-related hospitalization	15 (57.7%)
Any comorbidity present	13 (50.0%)
Any anatomical complexity	5 (19.2%)
Renal Doppler performed	22 (84.6%)
Procedure time (min)	79.61 ± 36.09
Contrast volume used (mL)	58.27 ± 30.08
Ablations	
Total (mean ± SD)	27.48 ± 9.82
Right renal artery (mean ± SD)	14.04 ± 5.29
Left renal artery (mean ± SD)	13.57 ± 6.75

HTN: hypertension; RDN: renal denervation; SD: standard deviation.

### BP control

**Figure 2:**
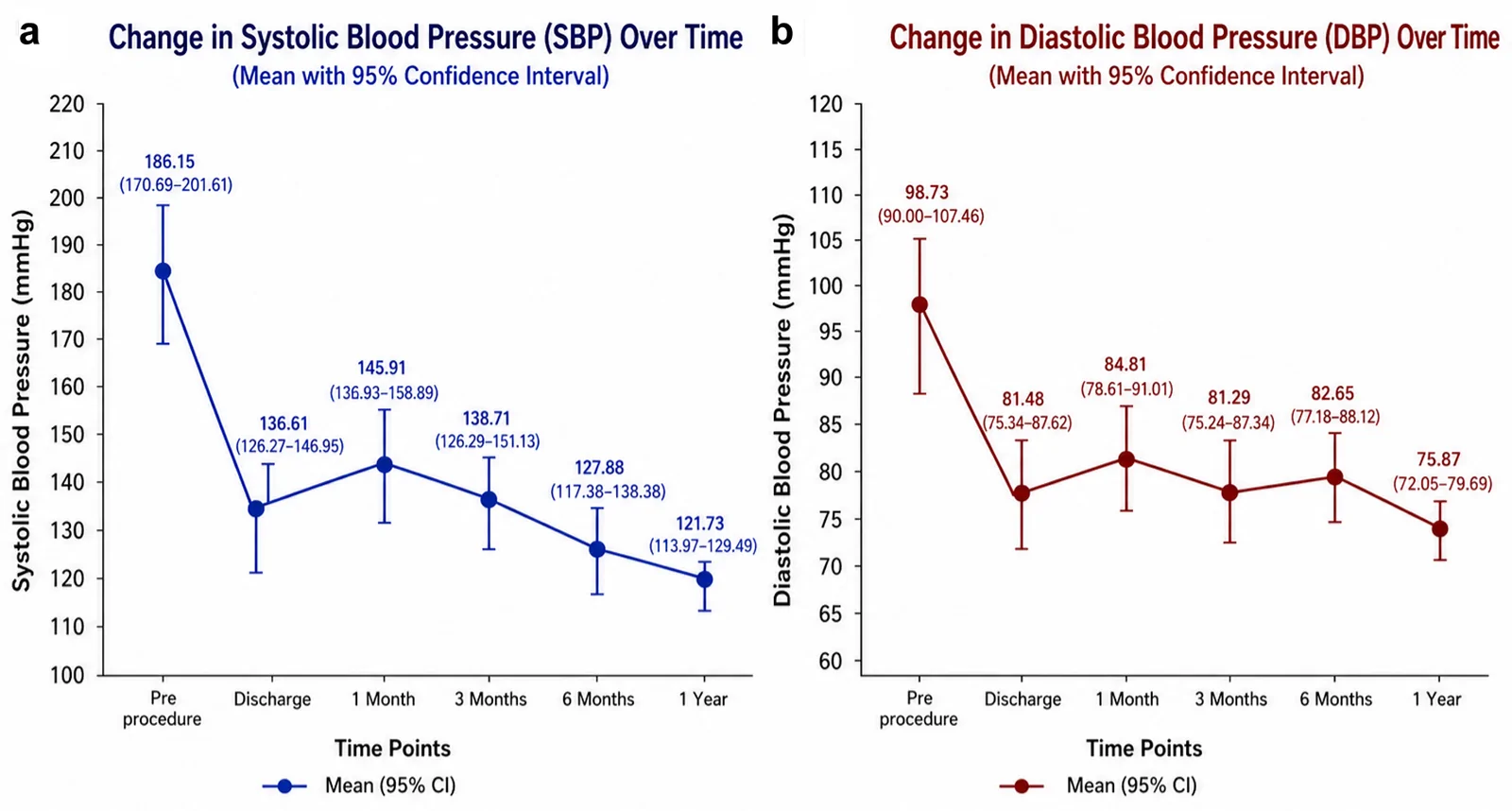
Changes in systolic (a) and diastolic (b) blood pressure following renal denervation.

**Figure 3:**
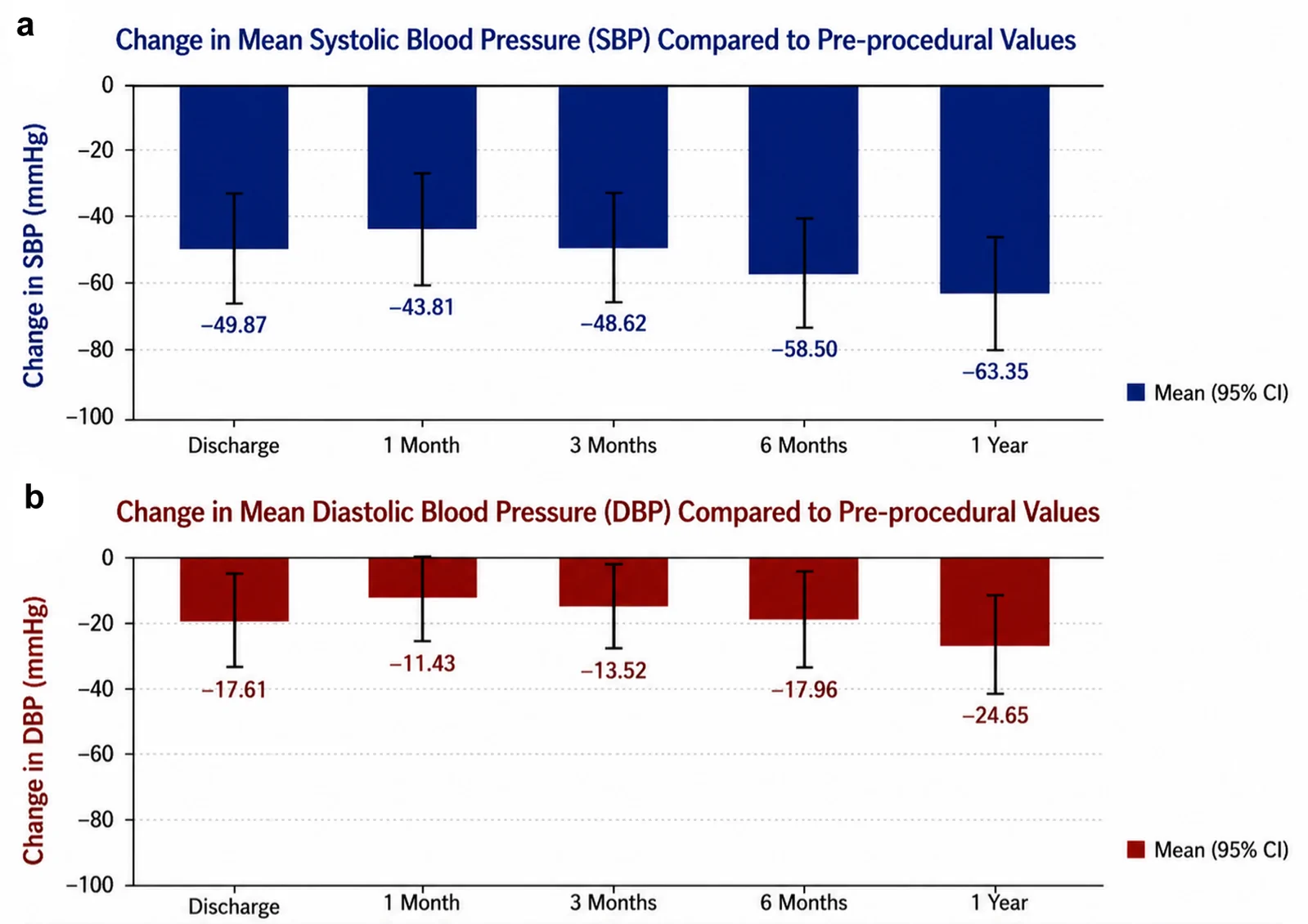
Mean change in systolic (a) and diastolic (b) blood pressure from baseline following renal denervation.

At baseline, the mean pre-procedural SBP was 186.2 ± 18.8 mm Hg and mean DBP was 98.7 ± 25.3 mm Hg, indicating markedly elevated BP prior to intervention. At discharge, there was a significant reduction in BP, with mean SBP decreasing to 136.6 ± 27.3 mm Hg (mean change −49.9 mm Hg; P < 0.001) and mean DBP decreasing to 81.5 ± 14.7 mm Hg (mean change −17.6 mm Hg; P = 0.003). At the 1-month follow-up, mean SBP was 145.9 ± 17.8 mm Hg and DBP was 84.8 ± 8.3 mm Hg, corresponding to reductions of −43.8 mm Hg (P < 0.001) and −11.4 mm Hg (P = 0.010), respectively, demonstrating a slight attenuation compared to discharge but remaining significantly lower than baseline. At 3 months follow-up, mean SBP further improved to 138.7 ± 16.4 mm Hg (mean change −48.6 mm Hg; P < 0.001), while mean DBP was 81.3 ± 10.1 mm Hg (mean change −13.5 mm Hg; P = 0.002), indicating sustained BP control. At 6 months, a more pronounced reduction in SBP was observed, with mean SBP decreasing to 127.9 ± 7.9 mm Hg (mean change −58.5 mm Hg; P < 0.001), while mean DBP was 82.7 ± 7.8 mm Hg (mean change −18.0 mm Hg; P = 0.002), with reduced variability suggesting improved stability of control. At 1 year, maximal BP reduction was achieved, with mean SBP further decreasing to 121.7 ± 10.5 mm Hg (mean change −63.4 mm Hg; P < 0.001) and mean DBP to 75.9 ± 4.7 mm Hg (mean change −24.7 mm Hg; P < 0.001) (Figs. 2 and 3).

### Medication burden

**Figure 4:**
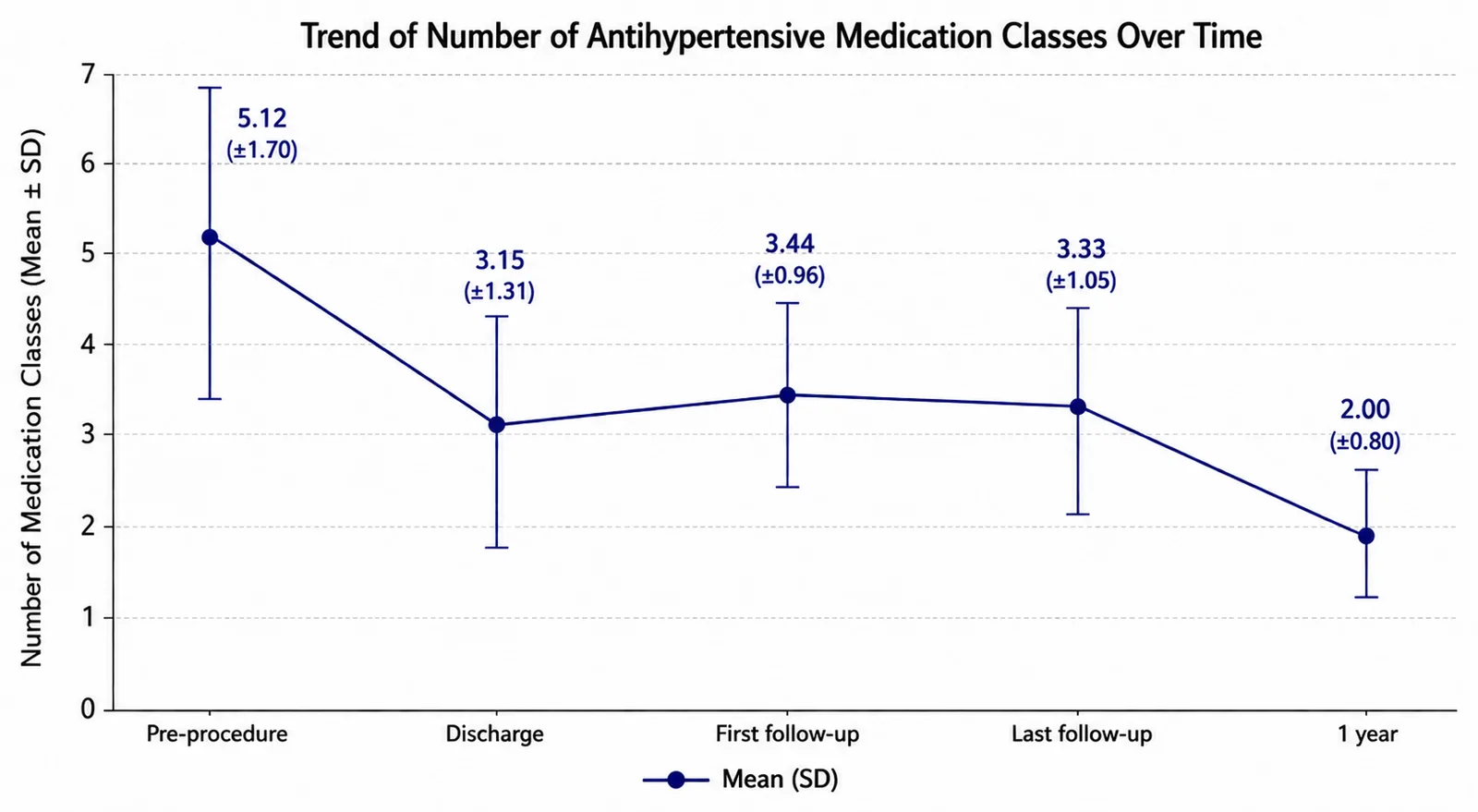
Reduction in antihypertensive medication burden following renal denervation.

The number of anti-hypertensive medication classes prescribed demonstrated a significant and progressive reduction over time. At baseline, patients were receiving a mean of 5.12 ± 1.70 medication classes. At discharge, this decreased to 3.15 ± 1.31, representing a significant reduction compared to baseline (P < 0.001). At 1 month follow-up, the mean number of medication classes was 3.44 ± 0.96 (P < 0.001 vs. baseline), and at 3 months follow-up it was 3.33 ± 1.05 (P < 0.001), indicating sustained reduction in pharmacological burden during early and intermediate follow-up. At 1 year, a further reduction was observed, with patients requiring a mean of 2.00 ± 0.80 medication classes, corresponding to a mean reduction of approximately −3.35 classes compared to baseline (P < 0.001). Overall, there was a marked and sustained decrease in anti-hypertensive medication burden over time, with the greatest reduction observed at 1 year (Fig. 4).

### Safety outcomes

No major periprocedural or long-term adverse events were observed. One patient developed a right thigh hematoma with an associated superficial femoral artery (SFA) leak, which was successfully managed with covered stent placement without further sequelae. No additional vascular complications, renal impairment, or device-related adverse events were noted during follow-up. Overall, RDN demonstrated a favorable safety profile in this cohort.

### Sub-group analysis

#### Sex-based analysis

A sex-based subgroup analysis demonstrated comparable baseline SBP between females (185.6 ± 23.3 mm Hg) and males (186.5 ± 16.2 mm Hg), whereas baseline DBP was higher in males (105.9 ± 27.5 vs. 87.2 ± 16.6 mm Hg). Both females and males demonstrated a substantial and progressive reduction in SBP following RDN. At discharge, SBP reduction was comparable between females (−49.2 mm Hg) and males (−50.3 mm Hg). At 1 month, a slightly attenuated reduction was observed, more pronounced in females (−47.3 mm Hg) compared to males (−41.2 mm Hg). Thereafter, a progressive decline was noted, with SBP reductions at 3 months (−54.6 vs. −45.3 mm Hg), 6 months (−57.6 vs. −59.7 mm Hg), and 1 year (−64.1 vs. −63.4 mm Hg) in females and males, respectively, indicating a sustained and comparable long-term effect across both sexes.

In contrast, DBP reduction demonstrated a differential pattern. At discharge, males showed a greater reduction than females (−21.7 vs. −11.2 mm Hg). This difference persisted at 1 month (−16.0 vs. −5.3 mm Hg) and 3 months (−16.8 vs. −10.4 mm Hg). At 6 months and 1 year, the magnitude of DBP reduction remained consistently higher in males (−23.1 and −28.9 mm Hg) compared with females (−7.3 and −15.4 mm Hg), suggesting a more pronounced diastolic response in males over time.

Both females and males demonstrated a significant reduction in antihypertensive medication burden following RDN. Baseline medication use was comparable between groups (females: 5.3 ± 1.83 vs. males: 5.0 ± 1.67 classes). At discharge, a significant reduction was observed in both females (3.25 ± 1.39) and males (3.08 ± 1.31; both P ≈ 0.001). This reduction was sustained at 1 month follow-up and 3 months follow-up in both groups (P < 0.05). At 1 year, medication burden further declined to 2.2 ± 1.03 classes in females and 2.0 ± 0.58 classes in males, representing an overall reduction of approximately three medication classes from baseline. Overall, the magnitude and trajectory of medication reduction were comparable between sexes.

#### Age-based subgroup analysis

Patients were stratified by age into < 60 years (n = 9) and ≥ 60 years (n = 14) groups. Patients aged ≥ 60 years had higher baseline SBP than those aged < 60 years (189.8 ± 19.6 vs. 179.2 ± 19.4 mm Hg), whereas baseline DBP was lower in the ≥ 60-year group (93.2 ± 15.9 vs. 107.1 ± 37.7 mm Hg).

Both age groups demonstrated significant reductions in SBP following RDN. In patients aged ≥ 60 years, SBP decreased from 189.8 ± 19.6 mm Hg at baseline to 149.5 ± 27.1 mm Hg at discharge (P < 0.001), 152.5 ± 13.9 mm Hg at 1 month (P < 0.001), 140.8 ± 15.9 mm Hg at 3 months (P < 0.001), 127.1 ± 6.2 mm Hg at 6 months (P < 0.001), and 120.7 ± 12.7 mm Hg at 1 year (P < 0.001). Similarly, in patients aged < 60 years, SBP decreased from 179.2 ± 19.4 mm Hg at baseline to 116.6 ± 17.4 mm Hg at discharge (P < 0.001), 139.7 ± 22.6 mm Hg at 1 month (P = 0.002), 134.6 ± 20.9 mm Hg at 3 months (P < 0.001), 125.6 ± 8.5 mm Hg at 6 months (P < 0.001), and 122.8 ± 5.6 mm Hg at 1 year (P < 0.001).

For DBP, patients aged ≥ 60 years showed significant reductions from 93.2 ± 15.9 mm Hg at baseline to 79.2 ± 12.0 mm Hg at discharge (P = 0.009), 82.5 ± 6.0 mm Hg at 1 month (P = 0.019), 77.8 ± 6.5 mm Hg at 3 months (P = 0.002), 80.3 ± 3.0 mm Hg at 6 months (P = 0.021), and 74.7 ± 4.3 mm Hg at 1 year (P = 0.036). In patients aged < 60 years, DBP decreased from 107.1 ± 37.7 mm Hg at baseline to 85.8 ± 20.0 mm Hg at discharge (P = 0.087), 89.3 ± 12.2 mm Hg at 1 month (P = 0.145), 87.0 ± 14.6 mm Hg at 3 months (P = 0.140), 86.4 ± 9.0 mm Hg at 6 months (P = 0.071), and 77.4 ± 5.1 mm Hg at 1 year (P = 0.036).

#### Patients with and without comorbidities

In the comorbidity-based subgroup analysis (n = 25), patients with comorbidities had higher baseline SBP (190.6 ± 15.3 vs. 181.8 ± 22.3 mm Hg) and DBP (103.3 ± 34.1 vs. 93.8 ± 11.5 mm Hg) compared to those without comorbidities. SBP decreased significantly in both groups at discharge (150.0 ± 31.2 mm Hg; P = 0.0002 vs. 124.3 ± 17.2 mm Hg; P < 0.001), with sustained reductions at 1 month (156.0 ± 14.0 vs. 136.4 ± 15.0 mm Hg; both P < 0.001) and 3 months (147.8 ± 16.0 vs. 129.7 ± 10.2 mm Hg; both P < 0.001). At 6 months, SBP was comparable (126.5 ± 7.8 vs. 127.9 ± 7.6 mm Hg; both P < 0.001), and remained significantly reduced at 1 year (119.6 ± 13.2 vs. 124.2 ± 4.9 mm Hg; both P < 0.001).

DBP reduction was significant in patients without comorbidities at discharge (76.5 ± 9.5 mm Hg; P = 0.0004) and 1 month (83.9 ± 9.0 mm Hg; P = 0.023), whereas it did not reach significance in those with comorbidities (86.7 ± 18.1 mm Hg; P = 0.081 and 85.5 ± 8.5 mm Hg; P = 0.054). By 3 months, DBP reduction became significant in both groups (79.8 ± 12.3 mm Hg; P = 0.021 vs. 83.2 ± 7.5 mm Hg; P = 0.013), and remained significant at 6 months (84.7 ± 8.5 mm Hg; P = 0.034 vs. 82.5 ± 4.4 mm Hg; P = 0.001) and 1 year (76.4 ± 4.9 mm Hg; P = 0.02 vs. 74.7 ± 4.2 mm Hg; P < 0.001) (Table 2).

**Table 2:** Longitudinal Changes in Systolic and Diastolic Blood Pressure Across Overall and Subgroup Analyses Following Renal Denervation

	Pre-procedure		Discharge		1-month follow-up		3-month follow-up		6-month follow-up		1-year follow-up	
	SBP	DBP	SBP	DBP	SBP	DBP	SBP	DBP	SBP	DBP	SBP	DBP
Overall BP (n = 26)	186.16 ± 18.75	98.73 ± 25.27	136.6 ± 27.35	81.47 ± 14.71	145.9 ± 17.82	84.81 ± 8.33	138.71 ± 16.4	81.28 ± 10.07	127.88 ± 7.87	82.65 ± 7.8	121.73 ± 10.46	75.87± 4.67
Females (n = 10)	185.6 ± 23.27	87.2 ± 16.58	138.12 ± 17.37	76.78 ± 10.07	140 ± 17.23	82.67 ± 7.30	133.23 ± 16.93	79.9 ± 10.37	128 ± 7.29	81.78 ± 4.71	121.5 ± 14.36	73.67 ± 3.67
Males (n = 16)	186.5 ± 16.17	105.93 ± 27.5	135.65 ± 32.82	84.5 ± 16.7	150.3 ± 17.6	86.41 ± 9.97	142.83 ± 15.41	82.33 ± 10.16	128.06 ± 8.01	83.21 ± 7.6	123.13 ± 5.43	77.43 ± 4.24
Age less than 60 years (n = 9)	179.2 ± 19.4	107.1 ± 37.7	116.6 ± 17.4	85.8 ± 20.0	139.7 ± 22.6	89.3 ± 12.2	134.6 ± 20.9	87.0 ± 14.6	125.6 ± 8.5	86.4 ± 9.0	122.8 ± 5.6	77.4 ± 5.1
Age more than 60 years (n = 14)	189.8± 19.6	93.2 ± 15.9	149.5 ± 27.1	79.2 ± 12.0	152.5 ± 13.9	82.5 ± 6.0	140.8 ± 15.9	78.27 ± 6.14	127.1 ± 6.2	80.3 ± 3.0	120.7 ± 12.7	74.7 ± 4.3
Family history of hypertension (n = 13)	181.77 ± 18.93	105 ± 32.33	127.91 ± 21.97	85.91 ± 18.01	143 ± 17.94	88.9 ± 9.18	142.4 ± 19.64	83.7 ± 12.86	125.38 ± 8.02	84.42 ± 7.72	122.31 ±5.55	76.33 ± 4.96
With comorbidities (n = 13)	190.61 ± 15.33	103.31 ± 34.01	150 ± 31.19	86.73 ± 18.10	156 ± 14	85.55 ± 8.55	147.82 ± 15.97	79.82 ± 12.30	126.46 ± 7.81	84.67 ± 8.47	119.62 ± 13.15	76.42 ± 4.94
Diabetes (n = 10)	191.7 ± 17.31	101.5 ± 38.94	151.11 ± 32.44	88.67 ± 19.62	154.25 ± 14.40	84.25 ± 5.28	142.66 ± 15.17	76.88 ± 5.94	124.4 ± 7.54	79.78 ± 6.67	118.5 ± 15.61	75.78 ± 5.02

BP: blood pressure; DBP: diastolic blood pressure; SBP: systolic blood pressure.

## Discussion

RF RDN has consistently demonstrated durable BP reduction across randomized sham-controlled trials and real-world registries, primarily conducted in Western populations [11]. These studies have included patients with resistant or difficult-to-control hypertension and varying comorbidity profiles, establishing RDN as a promising adjunctive therapy. However, the generalizability of these findings to the Indian population remains uncertain. Indian patients exhibit distinct clinical characteristics that may influence response to RDN, including earlier onset of hypertension, higher burden of metabolic risk factors, variable medication adherence, and differences in healthcare access and treatment patterns [12]. Despite these distinctions, there is a paucity of data evaluating the effectiveness of RDN in Indian cohorts.

To our knowledge, this represents one of the first real-world analyses of RF RDN from an Indian population. In this retrospective study of 26 patients with up to 1-year follow-up, we demonstrate significant and sustained reductions in BP, providing important region-specific evidence that complements existing global data.

The magnitude of BP reduction observed in the present cohort (−63.4 mm Hg SBP and −24.7 mm Hg DBP at 1 year) exceeds that reported in contemporary sham-controlled trials and registries conducted predominantly in Western populations. In SPYRAL HTN-OFF MED [13] and ON MED [14], as well as RADIANCE-HTN SOLO [15] and TRIO [16], reductions in ambulatory SBP were modest, typically in the range of −5 to −10 mm Hg. In contrast, earlier non–sham-controlled studies such as SYMPLICITY HTN-1 [17] and HTN-2 [18] reported larger reductions in office BP (approximately −27 to −32 mm Hg SBP and −10 to −12 mm Hg DBP at 6–12 months), although still lower than those observed in the present study. Importantly, data in Asian populations remain limited. In the Global SYMPLICITY Registry Korean cohort [19], office SBP decreased by −19.4 ± 17.2 mm Hg at 6 months and −27.2 ± 18.1 mm Hg at 12 months (both P < 0.001), confirming sustained BP reduction with a favorable safety profile.

Several factors may explain these differences by variations in study design and BP assessment methodology. Sham-controlled trials use ABPM and incorporate rigorous control of confounding factors such as medication adherence, regression to the mean, and placebo effects, resulting in more conservative but internally valid estimates of treatment effect. In contrast, earlier non–sham-controlled studies relied predominantly on office BP measurements, which are more susceptible to observer bias, Hawthorne effect, and variability, often leading to larger apparent reductions.

A recent study by Ratti et al [20] (2026) demonstrated that catheter-based RDN resulted in a significant reduction in BP among patients with RH, with mean SBP decreasing by 49.2 ± 12.1 mm Hg (from 175.1 ± 8.2 to 122.7 ± 13.0 mm Hg) and DBP by 12.2 ± 8.7 mm Hg (from 88.7 ± 7.1 to 71.5 ± 7.4 mm Hg) at 3-month follow-up. The procedure was also associated with a reduction in antihypertensive medication burden.

In this study, the greater magnitude of BP reduction is likely attributable to multiple factors. First, patients had higher baseline BP levels, reflecting a more severe hypertensive phenotype, which is a well-recognized predictor of greater response to RDN. Second, the real-world nature of this cohort may have allowed concurrent improvements in medication adherence and lifestyle modifications, both of which can augment BP reduction but are less apparent in tightly controlled trial settings. Third, procedural factors, including a higher number of ablations and more extensive circumferential and branch vessel treatment, may have resulted in more effective sympathetic denervation. Finally, unlike randomized sham-controlled trials that incorporate standardized run-in periods, ABPM, and rigorous control of potential confounding factors, our study relied on office BP measurements and routine clinical follow-up. Accordingly, regression to the mean, observer-related effects, and the absence of a sham-control group may also have contributed to the magnitude of the observed treatment effect. Prior registry data suggest greater long-term BP reduction in Asian cohorts compared with Western populations, potentially reflecting variations in sympathetic activity, salt sensitivity, or vascular physiology.

In the present study, RDN was associated with a substantial and progressive reduction in antihypertensive medication burden, with the mean number of medication classes decreasing from 5.12 ± 1.70 at baseline to 2.00 ± 0.80 at 1 year (P < 0.001), corresponding to a reduction of approximately three medication classes. This magnitude of de-escalation is notably greater than that reported in randomized trials. In the SPYRAL HTN-ON MED trial [14], baseline medication use was relatively low (1.8 ± 1.0 in the RDN group), and over follow-up, medication burden remained stable or modestly increased in the control arm, with the RDN group requiring fewer medications compared with sham at 12 months (3.9 ± 3.1 vs. 5.5 ± 5.2; P = 0.0004), but without a marked absolute reduction from baseline. Similarly, in SYMPLICITY [17–19] studies and registry data, medication regimens were generally maintained or only modestly adjusted due to protocol-driven treatment strategies, limiting the extent of medication reduction. Despite the low procedural volume at several participating centers, favorable clinical outcomes were observed across sites. This may reflect standardized procedural techniques, careful patient selection, and performance by experienced interventional cardiologists at tertiary referral centers.

### Limitations

This study is limited by its retrospective, observational design without a sham or control group, introducing potential bias and confounding. The multicenter design included a relatively small number of patients from each participating center, reflecting the early adoption of RDN in India. This may have introduced inter-center variability and limits assessment of center-specific procedural experience and outcomes. The small sample size limits statistical power and generalizability, particularly for subgroup analyses. BP assessment was based on office measurements without systematic ambulatory monitoring. In addition, medication adherence was not objectively assessed. Detailed data on laboratory investigations, other vitals, medication adherence, and dose adjustments were not available. Procedural variability across centers may have influenced outcomes. Follow-up was limited to 1 year, precluding assessment of long-term durability. Finally, the cohort was derived from tertiary centers, which may limit broader applicability. The subgroup analyses were exploratory and based on small sample sizes, limiting statistical power and precluding definitive conclusions. These findings should therefore be considered hypothesis-generating and require confirmation in larger prospective studies. The observed BP reduction may have been influenced by unmeasured confounders, including medication adherence, lifestyle modifications, regression to the mean, and the absence of a sham-control group.

Future directions should include large, multi-centric randomized sham-controlled trials in Indian populations to validate efficacy and address population-specific responses. The ongoing GSR DEFINE global registry, a prospective, multinational real-world study of RDN, has also been initiated in India with planned follow-up up to 5 years. This is also expected to provide important long-term data on durability, safety, and effectiveness in Indian patients, addressing a critical gap in region-specific evidence and enhancing the generalizability of global RDN outcomes.

### Conclusion

In this multicenter, real-world study from India, catheter-based RDN was associated with significant and sustained reductions in BP and antihypertensive medication burden over 1 year, with a favorable safety profile. These findings provide important region-specific evidence supporting the effectiveness of RDN as an adjunctive treatment for RH in routine clinical practice. However, given the retrospective design, small sample size, and absence of a sham-control group, larger randomized studies and long-term registry data are needed to confirm these findings and further define the role of RDN in Indian patients.

### Impact on daily practice

This multicenter real-world Indian study demonstrates that catheter-based RDN can achieve substantial and sustained BP reduction while significantly decreasing antihypertensive medication burden in patients with RH. The findings support the feasibility of incorporating RDN into routine clinical practice for carefully selected patients with difficult-to-control hypertension. These data also provide important region-specific evidence from India, where real-world outcomes and long-term experience with RDN remain limited.

## Data Availability

The de-identified data supporting the findings of this study are available from the corresponding author upon reasonable request and subject to institutional approvals, ethical restrictions, and applicable patient confidentiality requirements.

## Abbreviations

ABPM: ambulatory blood pressure monitoring
BP: blood pressure
DBP: diastolic blood pressure
ESC: European Society of Cardiology
ESH: European Society of Hypertension
GSR: Global SYMPLICITY Registry
ICMR: Indian Council of Medical Research
RADIANCE-HTN: Renal Denervation in Hypertension Trial
RDN: renal denervation
RF: radiofrequency
RH: resistant hypertension
SBP: systolic blood pressure
SD: standard deviation
SPYRAL HTN: Global Clinical Program of Renal Denervation
SYMPLICITY HTN: Symplicity Hypertension Trial
